# Experiences and perceptions of patients, caregivers, and healthcare professionals with long-acting injectable antipsychotics for the treatment of schizophrenia: qualitative results from the multinational ADVANCE study

**DOI:** 10.3389/fpsyt.2025.1645328

**Published:** 2025-12-09

**Authors:** Kelli R. Franzenburg, Rolf Hansen, Mark Suett, Ayelet Yaari, Martin Sergerie, Aviva Peyser Levin, Sigal Kaplan, Alma Gonzalez, Kameron Sedigh, Stephan Heres, Martha Sajatovic

**Affiliations:** 1 Global Medical Affairs, Teva Branded Pharmaceutical Products R&D, Inc., West Chester, PA, United States; 2 North America Medical Affairs, Teva Branded Pharmaceutical Products R&D, Inc., Parsippany, NJ, United States; 3 Global Medical Affairs, Teva UK Limited, Harlow, United Kingdom; 4 Global Medical Affairs, Teva Pharmaceutical Industries Ltd., Tel Aviv, Israel; 5 Medical Affairs, Teva Canada, Montreal,QC, Canada; 6 International Markets Medical Affairs, Teva Pharmaceutical Industries Ltd., Tel Aviv, Israel; 7 Global Health Economics and Outcome Research, Teva Pharmaceutical Industries Ltd., Netanya, Israel; 8 Syneos Health, New York, NY, United States; 9 Klinik Nord, kbo Isar-Amper-Kliniken Metropolregion Muenchen, and Technical University Munich TUM, München, Germany; 10 Case Western Reserve University School of Medicine, University Hospitals Cleveland Medical Center, Cleveland, OH, United States

**Keywords:** long-acting injectable antipsychotics, schizophrenia, healthcare professionals, patients, caregivers, setting of care, treatment preference

## Abstract

**Introduction:**

Schizophrenia imposes a substantial burden on individuals and society. Long-acting injectable antipsychotics (LAIs) improve adherence and reduce relapse and hospitalization rates compared with oral treatments for schizophrenia, yet LAI use varies globally. The qualitative interview portion of the global Attitudes DriVing regional differences in long-acting injectable ANtipsychotic utilization for schizophrenia among healthcare professionals (HCPs), patients, and CaregivErs (ADVANCE) study explored the patient journey from schizophrenia diagnosis to treatment, treatment goals, and experiences and perspectives on LAIs that may influence their use.

**Methods:**

ADVANCE included HCPs, patients, and caregivers from Australia, Canada, China, Germany, Israel, Spain, South Korea, and the United States. Eligible HCPs spent *≥*65% of their time providing direct patient care, managed an adult population of whom ≥10% have schizophrenia, and treated patients with second-generation LAIs. Patients with schizophrenia aged ≥18 years and caregivers of individuals living with schizophrenia who had experience with LAIs were eligible. Participants completed a 60-minute, semi-structured telephone interview.

**Results:**

Seventeen HCPs, 20 patients, and 19 caregivers completed interviews. HCPs reported that patients often follow a cycle of treatment and relapse, with inpatient care focused on acute symptoms management, while outpatient treatment was more likely to prioritize long-term quality of life. The most common HCP-reported barriers to LAI use were patient aversion to injections, logistical challenges, and patient trauma from prior forced injection. Two distinct treatment/disease management pathways emerged from patient interviews: 1) treatment early in symptom development with strong outpatient support, and 2) severe, acute episodes that required hospitalization. Patients initially treated in an outpatient setting were likely to accept LAIs, while those treated in inpatient settings often feared treatment, felt a lack of control, and were less likely to accept an LAI for long-term care. Caregivers had roles in disease management and focused more on patient quality of life rather than treatment management.

**Conclusions:**

Interactions between patients with schizophrenia and psychiatrists or psychiatric nurses vary depending on the care setting, which can influence the acceptance of LAIs. Initial schizophrenia presentation, family support, hospitalizations, trust in HCPs, and logistical challenges may all play a role in patient outcomes and perceptions of LAIs.

## 1 Introduction

Schizophrenia is a chronic, progressive disorder associated with significant disability and functional decline (1, 2). Globally, approximately 24 million people live with schizophrenia (3), and its prevalence is increasing (4). People living with schizophrenia often experience lifelong challenges, including alteration of thoughts, delusions, and hallucinations, that may affect everyday activities (5). Most people living with schizophrenia will experience multiple relapses, which exacerbate the risk of self-harm and impedes educational and occupational attainment (6). Schizophrenia can have a profound effect on a person’s quality of life (QoL) and on their families (2). Many people with schizophrenia have caregivers who play an important role and have varying responsibilities that may include assistance with daily living activities and emotional, financial, and social support (7). As a result of these responsibilities, the burden on the caregivers can be substantial (7, 8).

While oral antipsychotic medications (OAs) have been the mainstay of schizophrenia treatment, adherence to OAs is low among patients with schizophrenia (9, 10). Over the last decade there has been a substantial increase in the number and types of LAI formulations available to clinicians (11, 12). Long-acting injectable antipsychotics (LAIs) are associated with improved medication adherence, which leads to reduced relapse and hospitalization rates compared with OAs in people with schizophrenia (13). LAIs require less frequent dosing than OAs and are administered in a variety of care settings, allowing for closer adherence monitoring by the patient’s healthcare team and earlier identification if discontinuation or missed doses should occur.

There is emerging evidence for the use of LAIs earlier in treatment, including in patients with first-episode psychosis or early-stage schizophrenia, yet many healthcare professionals (HCPs) reserve LAIs for patients with a history of poor adherence and/or severe symptoms (13–15). LAI use tends to be higher among HCPs who are younger, see more patients with schizophrenia per month, and work in a community or academic setting (10, 16). Patients with schizophrenia who are more commonly prescribed an LAI include men, individuals with lower educational levels, or those with limited family resources (17–19). In France and the United States (US), people who are supported by a caregiver are more likely to be prescribed an LAI (18, 19).

LAI use varies across countries, though the underlying reasons for these differences remain poorly understood (9, 13, 19–21). No large-scale, global survey has been conducted to investigate factors influencing LAI adoption and use. Such a survey may yield insights on ways to increase LAI use and improve patient outcomes. Further understanding of patients’ pathways to treatment, treatment goals, disease maintenance, and perspectives on and experiences with LAIs is needed (20, 21).

The Attitudes DriVing regional differences in LAI ANtipsychotic utilization for schizophrenia among healthcare professionals (HCPs), patients, and CaregivErs (ADVANCE) study was a novel, multinational study that aimed to elucidate perceptions of LAIs and the impact on LAI use (22, 23). The ADVANCE study consisted of two stages: a detailed qualitative interview stage and a quantitative survey stage. Here, we present findings from detailed qualitative interviews with people living with schizophrenia, caregivers of people with schizophrenia, and HCPs from the ADVANCE study.

This portion of the study aimed to better understand the 1) patient’s journey with schizophrenia from initial diagnosis to treatment and maintenance, 2) current experiences with schizophrenia, treatment goals for patients and caregivers, and qualities in HCPs that influence treatment decisions, and 3) HCPs’, patients’, and caregivers’ experiences and perspectives on LAIs, including the initial LAI recommendation and decision, and perceived advantages and disadvantages of LAIs.

## 2 Materials and methods

### 2.1 HCP participants: psychiatrists and psychiatric nurses

Eligible HCPs were psychiatrists and psychiatric nurses who spent ≥65% of their time providing direct patient care, managed an adult patient population of whom ≥10% have a schizophrenia diagnosis, and reported treating patients with second-generation LAIs in the last 12 months. Participants were located in Australia, Canada, China, Germany, Israel, Spain, South Korea, or the US. The desired sample was one HCP type (i.e., one psychiatrist and one nurse) per country, though this was flexible based on knowledge gaps and recruitment success. Panels maintained by Capvision, a global expert network, and publicly available lists were used to initially identify psychiatrists and psychiatric nurses in the included countries. These lists identified potential participants through professional networks such as LinkedIn, complemented by medical publication databases. Individuals identified through these panels and lists were contacted via targeted outreach within specific therapeutic areas and geographies.

Identified HCPs who responded to the initial outreach were invited to complete a 20-minute internet interview screening questionnaire that included questions about location, role, clinical practice characteristics (e.g., setting, number of patients managed), and experience with LAIs. Respondents were solely qualified based on their screening responses. While psychiatrists from South Korea were surveyed, psychiatric nurses could not be recruited in South Korea due to local compliance restrictions.

### 2.2 Patient and caregiver participants

Eligible patients were aged ≥18 years, diagnosed with schizophrenia, treated with an antipsychotic medication for schizophrenia, and either had experience with or had been previously recommended LAI treatment by their physician. Eligible caregivers played a role in the management and treatment of an individual with schizophrenia who had experience either being treated with an LAI antipsychotic or had been recommended an LAI by their physician. The individual with schizophrenia cared for by the caregiver must have met the inclusion criteria for patients outlined above. Caregivers were not linked or associated with the patient participants in this study. Patients and caregivers were located in Australia, Canada, China, Germany, Israel, Spain, South Korea, or the US. A larger number of patients and caregivers were recruited compared with HCPs, given the limited published research on their perspectives.

In Australia, Canada, Germany, Israel, Spain, and the US, a vendor used social and digital strategies and social community groups to effectively identify and recruit patients and caregivers. In China, patients and caregivers were recommended by HCPs in a vendor’s panel. In South Korea, patients and caregivers were recommended by HCPs or were identified from a vendor’s local database. To determine eligibility, a 20-minute internet interview screening was completed with patients with schizophrenia and caregivers of individuals with schizophrenia.

### 2.3 Interview procedures

Interviews were conducted by researchers via video or audio call. There was no prior relationship between the interviewer and the person interviewed. Each interview lasted 60 minutes. HCP interviews were conducted from May 2023 to July 2023 and patient and caregiver interviews were conducted from June 2023 to November 2023.

A semi-structured interview guide developed for this study was used for each interview. A global research approach was used to develop interview questions to understand variables potentially impacting LAI use at the country level. The interview questions were developed based on 1) findings of a literature review to identify potential barriers and drivers to LAI use, 2) findings of a literature review on the beliefs of patients with schizophrenia and caregivers of individuals with schizophrenia and potential barriers and drivers of LAI use, and 3) a country-level market assessment of LAI use and systemic variables that potentially impact LAI use. The interview questions were refined through review and iterative study team discussions.

Separate interview guides were developed for HCPs, patients, and caregivers. The HCP interview consisted of questions related to the HCP’s background and patient population, treatment approach for people with schizophrenia, and experiences and perceptions of LAIs. For patients, interview questions focused on the patient’s history of schizophrenia, current experience with schizophrenia, and experiences with and perceptions of LAIs. For caregivers, the interview questions were related to the caregiver’s individual with schizophrenia, that individual’s history with schizophrenia, and experiences with and perceptions of LAIs. The interview guides are included in Supplementary Material.

### 2.4 Interview analysis

All interviews were recorded and were transcribed verbatim with the exception of the removal of potentially personally identifying information. During and after each interview, notes were taken on the participant’s answers to the questions. The interview recordings were consulted to fill gaps in any of the notes. The notes across all interviews were reviewed and analyzed. The written details of each interview were categorized, coded, and quantified accordingly based on specific words, themes, and concepts within the data. A specific analytic framework was not used as part of this study.

## 3 Results

### 3.1 HCP participants: psychiatrists and psychiatric nurses

#### 3.1.1 Participant characteristics

Of the 1250 HCPs initially identified and contacted, 31 were screened, and 17 were invited to participate in the full interview. A large number of invitations to participate were sent out and the study team screened and eventually consented those who were the first to respond to the initial contact and who appeared to fit study entry criteria. The 10 interviewed psychiatrists (7 male, 3 female) and 7 interviewed psychiatric nurses (2 male, 5 female) were from Australia (*n* = 2), Canada (*n* = 2), China (*n* = 3), Germany (*n* = 2), Israel (*n* = 3), South Korea (*n* = 1), Spain (*n* = 2), and the US (*n* = 2). Psychiatrists had an average of 18 years of experience (range: 4 to 40 years) and psychiatric nurses had an average of 17 years of experience (range: 4 to 32 years). The psychiatrists and psychiatric nurses who were interviewed most often practiced in a hospital-based outpatient clinic (45% and 20%, respectively), an inpatient psychiatric ward (24% and 28%), a community mental health center/psychiatric outpatient clinic (19% and 47%), or independent/private practice or office (12% and 5%) (**Table 1**).

**Table 1:** HCP (*N* = 17) characteristics.

HCP type	Years of experience	LAI use (%)	Primary setting of care
Australia			
Psychiatrist	10	40	Hospital-based OP clinic
Psychiatric nurse	32	5	CMHC/Psych OP clinic
Canada			
Psychiatrist	27	40	Inpatient psych ward
Psychiatric nurse	28	50	Inpatient psych ward
China			
Psychiatrist	40	10	Inpatient psych ward
Psychiatrist	34	10	Hospital-based OP clinic/Inpatient psych ward
Psychiatric nurse	6	5	Inpatient psych ward
Germany			
Psychiatrist	10	20	Hospital-based OP clinic
Psychiatric nurse	4	20	Hospital-based OP clinic
Israel			
Psychiatrist	6	30	CMHC/Psych OP clinic
Psychiatrist	6	10	CMHC/Psych OP clinic
Psychiatric nurse	10	30	Inpatient psych ward
South Korea			
Psychiatrist	10	30	Hospital-based OP clinic
Spain			
Psychiatrist	4	30	Hospital-based OP clinic
Psychiatric nurse	25	40	CMHC/Psych OP clinic
United States			
Psychiatrist	35	50	CMHC/Psych OP clinic/Inpatient psych ward
Psychiatric nurse	11	60	CMHC/Psych OP clinic

CMHC, community mental health center; OP, outpatient; psych, psychiatric.

#### 3.1.2 Treatment objectives and decision-making

**Figure 1:**
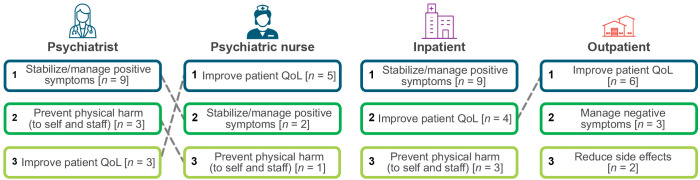
HCP-reported treatment objectives. HCP, healthcare professional; QoL, quality of life.

The top combined HCP-reported treatment objectives for their patients with schizophrenia were to stabilize and manage positive symptoms of schizophrenia (65%), improve patient QoL (47%), prevent physical harm to self and staff (24%), manage negative symptoms (24%), and reduce side effects (12%) (**Figure 1**). Highlighting the importance of stabilizing and managing positive symptoms as well as improving quality of life, one HCP stated, “We are so focused on psychotic functions because we’re afraid they’ll do something really bad, but they want just to connect again with the world.” When stratified by type of HCP, psychiatrists most often reported their treatment objectives were to stabilize and manage positive symptoms (90%), prevent physical harm to self and staff (30%), and improve patient QoL (30%). Psychiatric nurses most often reported that their treatment objectives were to improve patient QoL (71%), stabilize and manage positive symptoms (29%), and prevent physical harm to self and staff (14%).

The top combined HCP-reported treatment objectives varied by setting of care (**Figure 1**). For inpatient settings of care, the top three HCP-reported treatment objectives were to stabilize and manage positive symptoms (53%), improve patient QoL (24%), and prevent physical harm to self and staff (18%). Conversely, improvement of patient QoL (35%), management of negative symptoms (18%), and reduction of side effects (12%) were the top three HCP-reported treatment objectives in outpatient settings of care.

In Australia, Canada, and Germany, HCPs reported full, paid coverage for patient management in a public inpatient setting. HCPs also reported that, in these countries, some patients will seek inpatient care in the emergency department rather than in an outpatient care facility with a community psychiatrist due to the cost discrepancy between seeking care in an inpatient versus outpatient facility. In Australia, Canada, China, Germany, and Israel, HCPs reported a lack of community psychiatrists in outpatient care, resulting in long wait times for patients seeking care with a psychiatrist.

Psychiatrists drove most treatment discussions in an inpatient setting with the objective to stabilize the patient and control positive symptoms. It was noted in Australia, Canada, and Israel that general practitioners may take the lead role in treatment discussion for outpatients. However, general practitioners will often need to consult a psychiatrist if treatment change is necessary. Psychiatric nurses reported low to medium influence on treatment discussions in both the inpatient and outpatient settings.

#### 3.1.3 LAI use

Psychiatrists reported mean LAI use of 27%, ranging from a low of 10% in China and Israel to a high of 50% in the US. Psychiatric nurses reported mean LAI use of 30%, ranging from 5% in China and Australia to 60% in the US. The reported LAIs used included haloperidol (47%), paliperidone palmitate (47%), aripiprazole (35%), risperidone (29%), olanzapine (18%), and zuclopenthixol (12%).

When describing their general view (i.e., mindset) regarding when to use an LAI, 40% of psychiatrists reported that they viewed LAIs as reserved for patients with adherence issues to oral medication (i.e., “adherence reserved”), 30% reported that they actively use LAIs as early as possible for their patients (i.e., “early LAI users”), and 30% indicated that they viewed LAIs as reserved for patients with more severe symptoms or for use after other treatments have failed (i.e., “severity reserved”). The nurses self-identified as “severity reserved” (43%), “adherence reserved” (29%), or “early LAI users” (29%).

**Figure 2:**
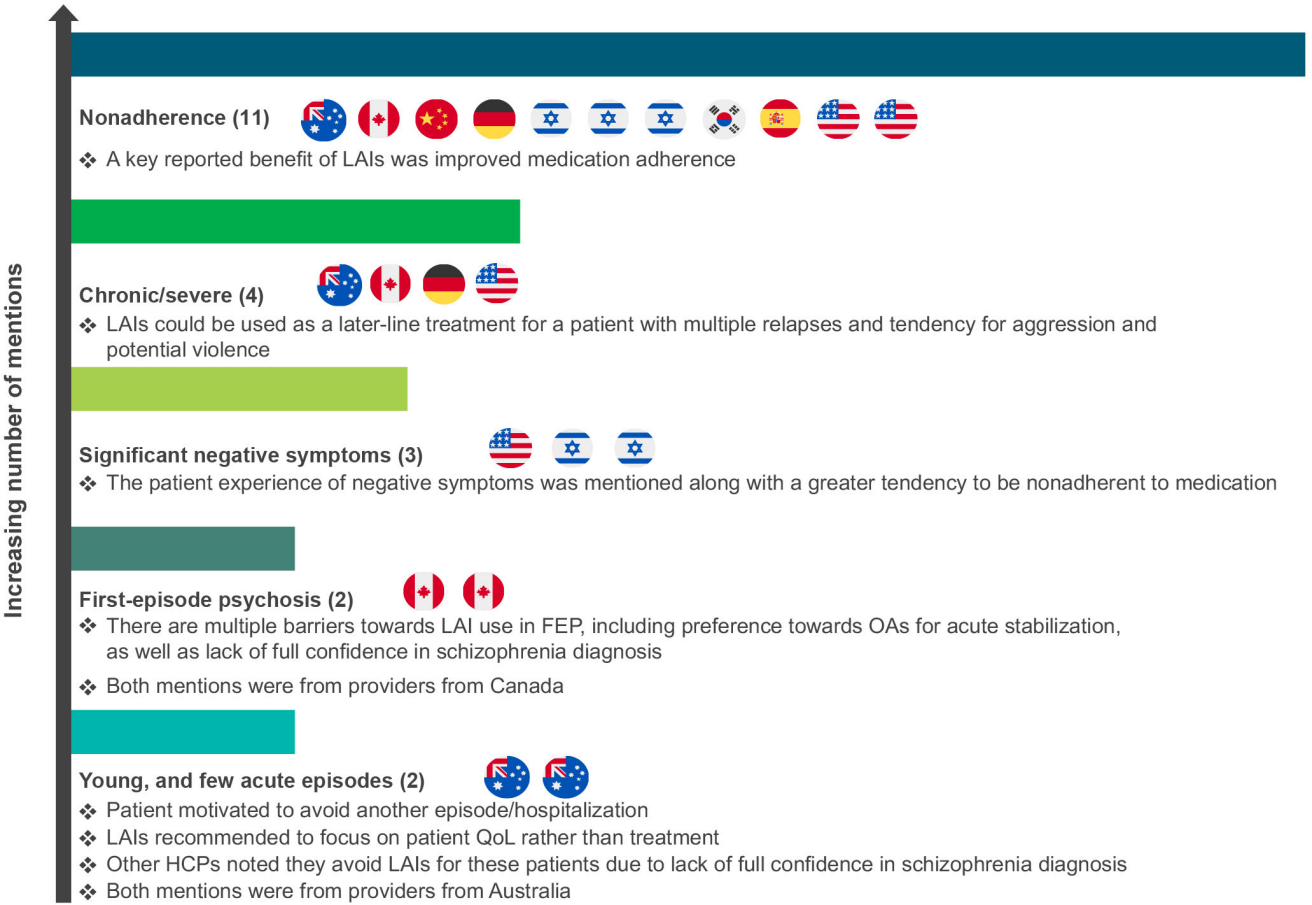
HCP-reported patient profiles for LAI use (number of mentions).^a^ FEP, first-episode psychosis; HCP, healthcare professional; LAI, long-acting injectable antipsychotic; OA, oral antipsychotic; QoL, quality of life. ^a^Each flag represents the country in which the HCP practices and who reported for that specific profile.

Among HCPs, there was a lack of consensus about the ideal patient profile for LAIs (**Figure 2**). Most HCPs believed that LAI use in younger patients or those with less severe disease should be limited. Two HCPs in Australia supported LAI use in younger patients with few acute episodes, with one stating, “LAIs keep them engaged in treatment long-term while figuring out housing, education, employment.” Two HCPs in Canada supported LAI use in patients with first-episode psychosis.

#### 3.1.4 Barriers to LAI use

Psychiatrists’ perceptions of barriers to LAI use included patient aversion to injections (70%), logistical challenges (60%), trauma from prior forced injection (50%), cost (40%), patient fear of loss of control (30%), and stigma (30%). For psychiatric nurses, patient trauma from prior forced injection (43%), logistical challenges (43%), lack of product availability (29%), cost (14%), patient aversion to injection (14%), and stigma (14%) were reported perceptions of barriers to LAI use. Some psychiatric nurses noted negative experiences relating to being the ones administering the LAI injections. While HCP-reported perceived barriers included limited availability of LAIs, a lack of available LAIs was primarily reported in South Korea and China, where only one to three LAIs are available. Cost was reported as the key barrier to LAI adoption in China.

### 3.2 Patients

#### 3.2.1 Participant characteristics

Of the 509 patients and caregivers initially identified and contacted, 80 patients responded to the initial outreach and were screened, 42 were invited to participate in the interviews, and 20 completed the full interview. The mean age at diagnosis was 27 years, mean disease duration was 11 years, and 60% were female (**Table 2**). When considering employment and living conditions, 60% of patients reported that they were unemployed or receiving disability payments and 35% were living independently. The majority of participants (90%) were initially diagnosed in an inpatient setting; the remaining 10% were diagnosed at a young age by a community psychiatrist.

**Table 2:** Patient (*N =* 20) demographics.

Sex	Age at diagnosis	Disease duration	Employment status	Caregiver status	Current LAI status (LAI type)
Australia					
Male	17	18	Receiving disability	None	Past LAI use (risperidone, flupentixol)
Male	50	5	Receiving disability	None	Current LAI use (paliperidone)
Female	34	3	Unemployed	Spouse	Current LAI use (not available)
Female	27	16	Receiving disability	None	Past LAI use (not available)
Canada					
Female	45	12	Employed	Child	Current LAI use (paliperidone)
China					
Female	27	19	Receiving disability	Parent	Never used LAIs
Female	33	5	Student	Parent	Current LAI use (olanzapine)
Female	13	6	Receiving disability	Parent	Past LAI use (not available)
Male	33	3	Receiving disability	Parent	Past LAI use (not available)
Germany					
Male	25	13	Unemployed	None	Current LAI use (haloperidol)
Israel					
Female	19	13	Employed	None	Past LAI use (olanzapine)
Male	28	7	Employed	None	Current LAI use (zuclopenthixol)
Male	26	19	Employed	None	Current LAI use (zuclopenthixol)
Male	37	20	Employed	None	Past LAI use (haloperidol, paliperidone)
South Korea					
Female	25	15	Unemployed	None	Current LAI use (not available)
Female	28	10	Unemployed	None	Current LAI use (not available)
Female	28	8	Unemployed	Parent	Current LAI use (not available)
Male	19	21	Receiving disability	Spouse	Current LAI use (not available)
Spain					
Female	20	5	Student	Parent	Current LAI use (paliperidone; past: aripiprazole)
United States					
Female	20	5	Student	Parent	Current LAI use (fluphenazine; past: paliperidone)

LAI, long-acting injectable antipsychotic.

Patients described their current treatment goals as mostly social (*n* = 9) or employment (*n* = 7) goals. Other reported treatment goals included reducing medication/side effects (*n* = 3), activities of daily living (*n* = 3), positive symptom control (*n* = 2), and education (*n* = 2).

#### 3.2.2 Treatment history

Among patients (*n* = 18; 90%) who could recall the name of the antipsychotic they were initially treated with upon schizophrenia diagnosis, 17 were treated with OAs and one received an LAI. The OAs most commonly reported as initial treatment were quetiapine and aripiprazole. Most patients (65%) reported currently receiving LAI treatment and 35% (*n* = 7) of patients reported prior LAI use. A wide range of LAIs were reported, with paliperidone LAI (*n* = 3) most mentioned.

LAIs were often first introduced by the patient’s physician (90%) and in an inpatient setting (70%). Patients expressed a desire for continuity of care with a trusted HCP who would understand their needs and optimize care of their unique situation.

**Figure 3:**
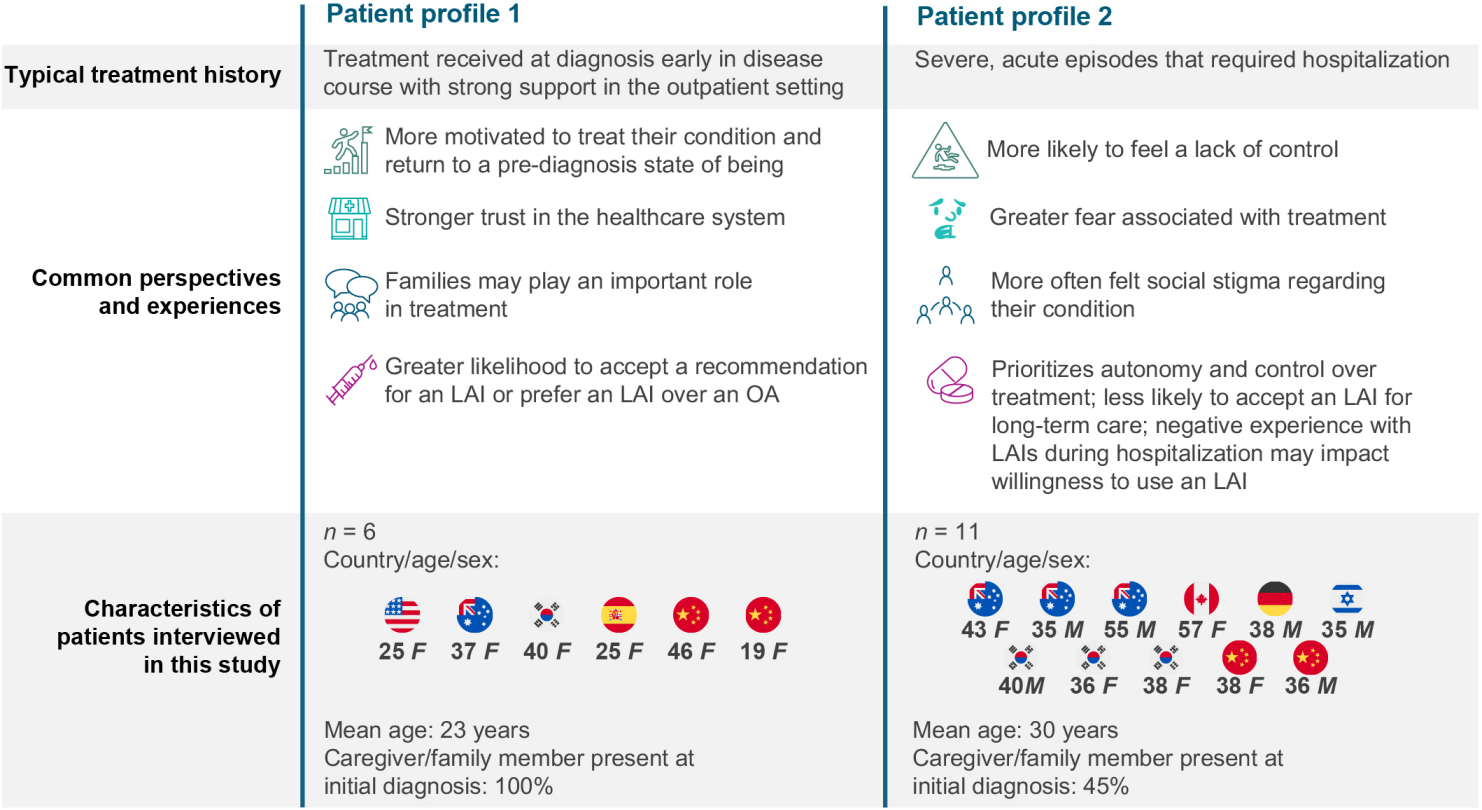
Two profiles emerged for patients with schizophrenia. F, female; LAI, long-acting injectable antipsychotic; M, male; OA, oral antipsychotic.

Patients had two distinct treatment/disease management profiles. Patients who presented early in symptom development and were treated at diagnosis with strong support in outpatient settings with no or a short stint in the hospital were more likely to prefer an LAI over an OA (**Figure 3**, Profile 1). Conversely, patients who presented with more severe acute episodes that required hospitalization were more likely to refuse an LAI than an OA (**Figure 3**, Profile 2).

#### 3.2.3 Perceptions of LAIs

**Figure 4:**
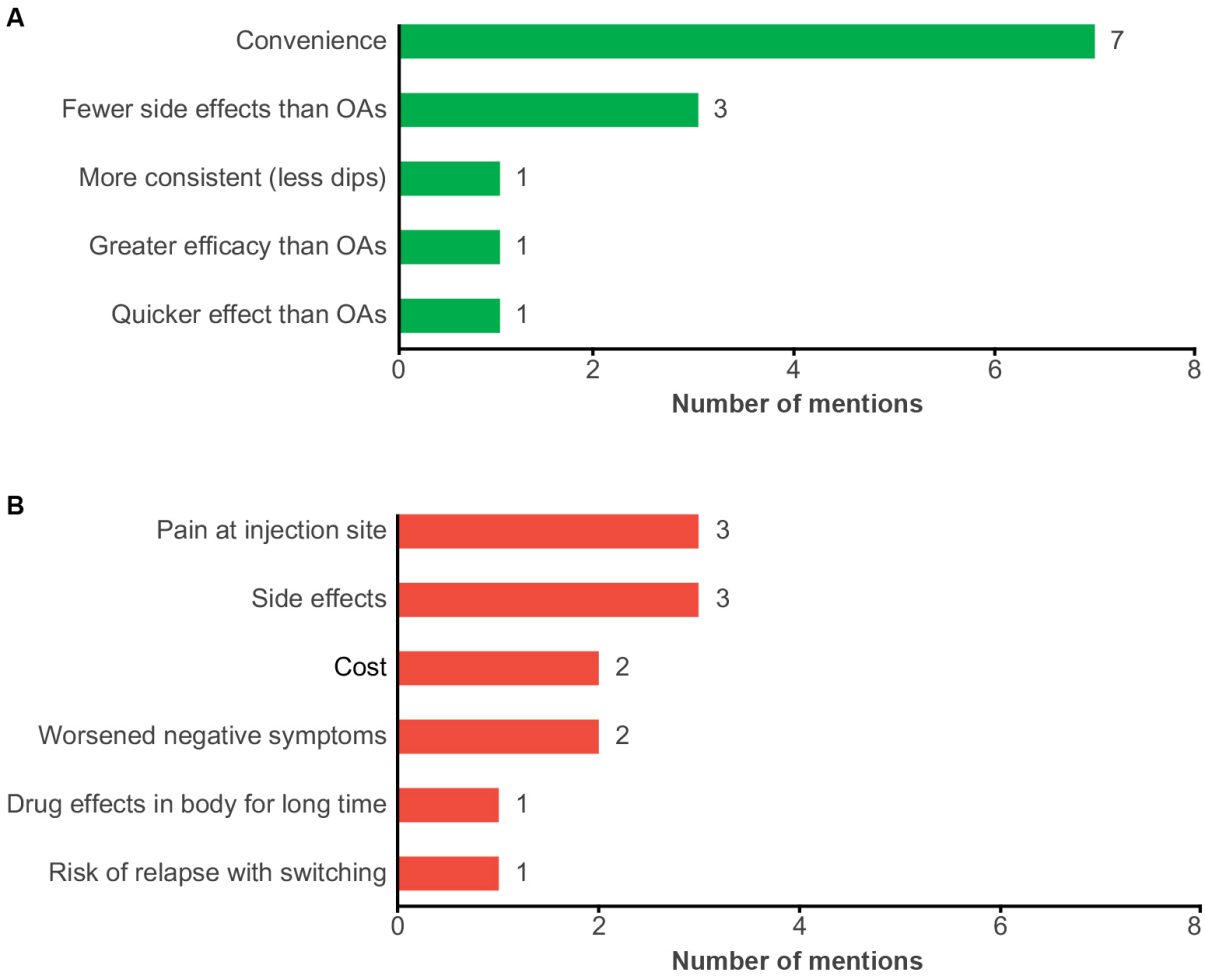
Patient-reported perceptions of LAIs with **(A)** perceived benefits and **(B)** perceived concerns. LAI, long-acting injectable antipsychotic; OA, oral antipsychotic.

Patients most commonly reported that they perceived the advantage of LAIs to be convenience (*n* = 7) and fewer side effects compared with OAs (*n* = 3). Additionally, patients reported that, compared with OAs, their treatment was more consistent (i.e., fewer symptomatic dips [*n* = 1]), and had greater efficacy (*n* = 1) and quicker onset of drug effects (*n* = 1) (**Figure 4A**).

The most commonly expressed concerns with LAIs were pain at the injection site (*n* = 3) and side effects (*n* = 3). Patients also reported concerns with cost (*n* = 2), worsening negative symptoms (*n* = 2), drug effects in body for a long time (*n* = 1), and risk of relapse with switching (*n* = 1) (**Figure 4B**). The two patients who expressed concerns related to cost were located in Israel and South Korea. One patient reported that they were unable to try an LAI due to cost.

### 3.3 Caregivers

#### 3.3.1 Participant characteristics

Among the 34 caregivers screened, 23 were invited to participate, and 19 completed the full interview. Among interviewed caregivers, 74% were family members or spouses of the person with schizophrenia. The mean years of experience as a caregiver to the person with schizophrenia was 7 years; 21% had been providing care for <5 years, 58% for 5–10 years, and 21% for >10 years (**Table 3**).

**Table 3:** Caregiver (*N* = 19) demographics.

Relationship to patient	Years as caregiver	Patient current LAI status
Australia		
Friend	8	Current LAI use
Sibling	5	Current LAI use
Parent	5	Past LAI use
Spouse	7	Current LAI use
Canada		
Professional	3	Current LAI use
China		
Sibling	3	Never used LAIs
Child	7	Current LAI use
Parent	10	Past LAI use
Parent	6	Past LAI use
Germany		
Parent	20	Past LAI use
Israel		
Parent	6	Past LAI use
Spouse	14	Current LAI use
Professional	8	Past LAI use
South Korea		
Spouse	30	Current LAI use
Sibling	10	Current LAI use
Professional	2	Current LAI use
Professional	3	Current LAI use
Spain		
Parent	23	Current LAI use
United States		
Child	8	Never used LAIs

LAI, long-acting injectable antipsychotic.

More than half (53%; *n* = 10) of caregivers cared for persons who were initially diagnosed with schizophrenia in an inpatient setting. Caregiver presence at initial diagnosis varied and depended on the relationship with the person with schizophrenia. Caregivers (60%; *n* = 6) who were present at initial diagnosis were either parents (30%) or siblings (30%).

The relationship between the caregiver and the person living with schizophrenia often changed over time. 6 of 9 (67%; 5 were children, siblings, or friends of the person living with schizophrenia) caregivers reported improvements and changing dynamics in the relationship that have brought them closer over time. 3 of 9 (33%) caregivers (2 parents, 1 spouse) reported continual conflict that has led to a worsening relationship with the person with schizophrenia.

#### 3.3.2 Caregiver roles

Caregiver responsibilities varied widely and included providing medication reminders, driving a person with schizophrenia to appointments, documenting and discussing medication side effects, initiating treatment discussions, and playing a vital role in providing emotional, financial, and logistical support.

Regarding treatment decisions, 21% of caregivers reported being somewhat involved in treatment decisions, 42% reported being involved, 26% reported being very involved, and 11% stated that they make treatment decisions for the person with schizophrenia. When considering treatments, caregivers emphasized the patient’s QoL along with symptom management as important factors in their decision.

#### 3.3.3 Caregiver perspectives on LAIs

Current and prior LAI use was reported for 58% (*n* = 11) and 32% (*n* = 6) of patients being cared for, respectively. Nearly all caregivers interviewed (95%) reported the patient’s physician as the one who initiated an LAI conversation. One caregiver who reported initiating LAI discussions had learned about LAIs from other patients in the hospital. The majority of caregivers (89%) were unaware of LAIs before the initial LAI conversation.

**Figure 5:**
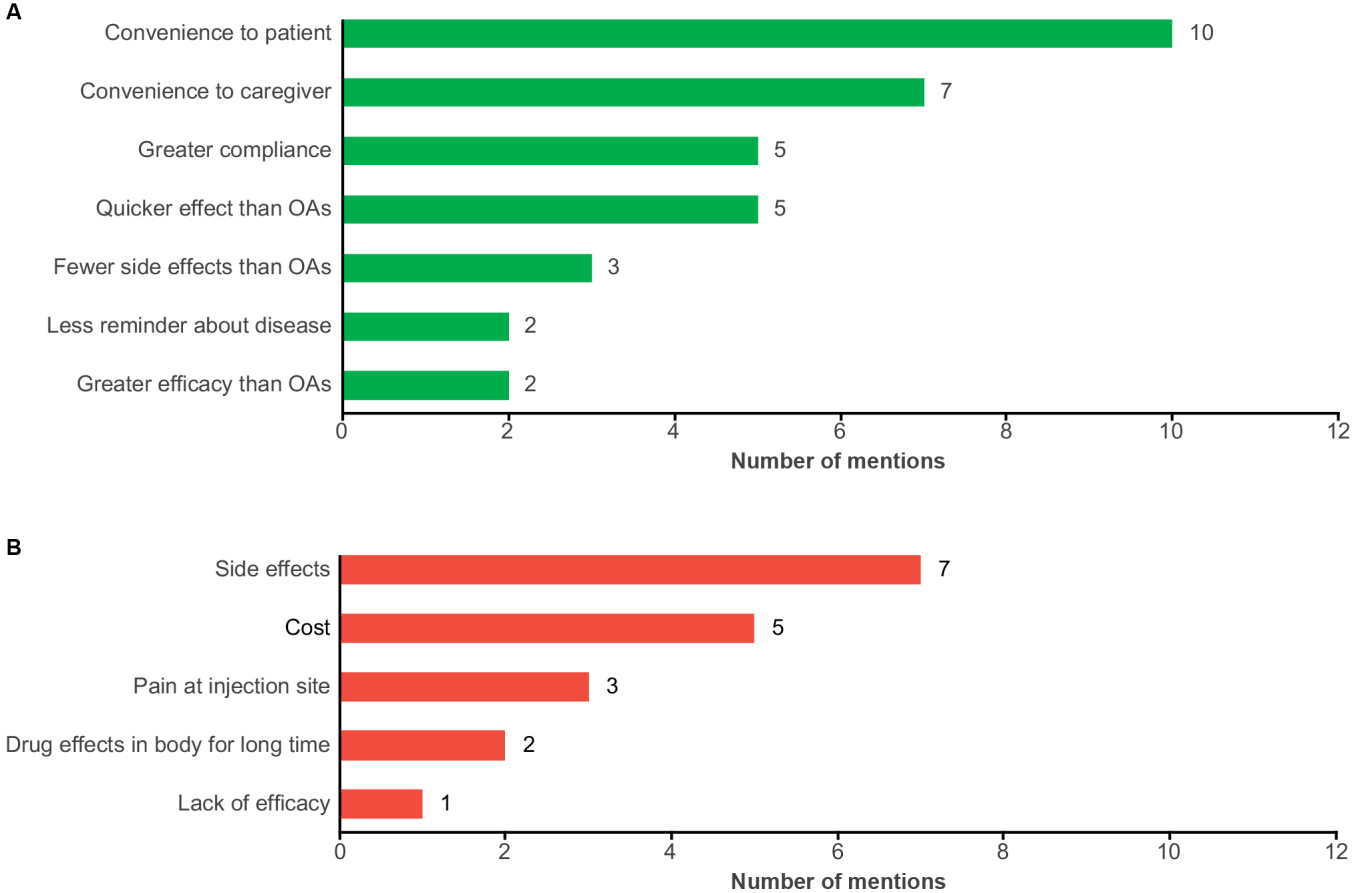
Caregiver-reported perceptions of LAIs with **(A)** perceived benefits and **(B)** perceived concerns. LAI, long-acting injectable antipsychotic; OA, oral antipsychotic.

Most caregivers reported that the advantages of LAIs included convenience to both the patient (53%) and the caregiver (37%). Additionally, greater adherence (26%), quicker effects than OAs (26%), fewer side effects (16%), fewer reminders of the disease (11%), and greater efficacy than OAs (11%) were mentioned (**Figure 5A**). For example, one caregiver stated, “With the injections, they don’t have to think about it. Daily pills are a reminder that they are not doing well.”

Side effects (37%) and cost (26%) were the most common concerns about LAIs; pain at injection site (16%), long-term drug effects (11%), and lack of efficacy (5%) were also reported concerns (**Figure 5A**). When caregivers were asked to describe their concerns about LAI use, side effects were identified due to lack of information and education about LAI treatment. Caregivers who reported cost as a concern were from Israel (*n* = 2), China (*n* = 2), and Canada (*n* = 1).

#### 3.3.4 Unmet needs

Caregivers most often reported needing more educational resources about treatment options for patients with schizophrenia (32%). Most caregivers reported that they had not received any information from the patients’ HCPs and that they had to do their own research to uncover and understand the best treatment options for the person with schizophrenia they cared for. Other caregiver-reported needs included caregivers support group (16%), wellness support (e.g., therapy) for the caregiver (16%), and quicker access to professionals (5%).

## 4 Discussion

**Figure 6:**
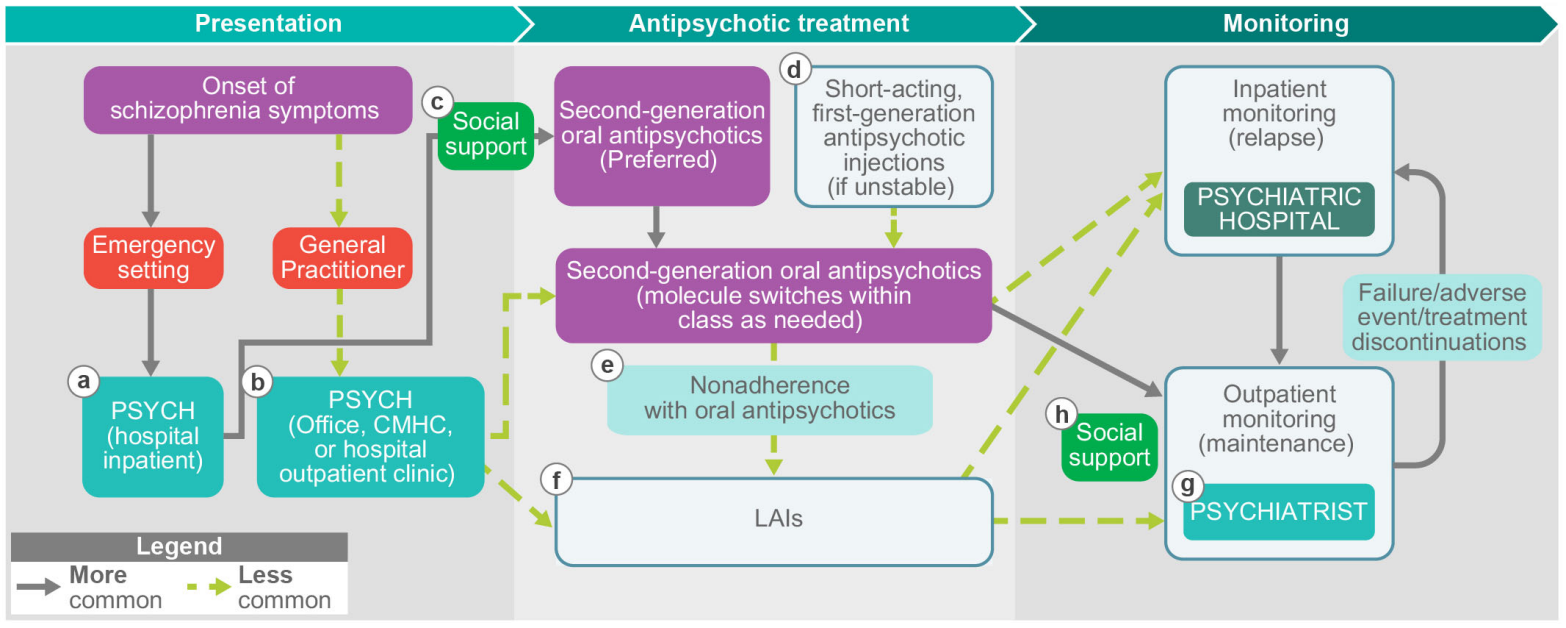
A conceptual journey from the onset of schizophrenia symptoms through treatment and monitoring. CMHC, community mental health center; HCP, healthcare professional; LAI, long-acting injectable antipsychotic; psych, psychiatrist. **(a)** Inpatient hospitalization experience may result in patients associating LAIs with loss of autonomy or pain, or there is a general lack of trust in HCPs. **(b)** A patient who presents early to the outpatient setting may be more likely to be involved in their treatment decision and recommended an LAI. **(c)** The presence of social support may be linked to greater likelihood of HCP LAI recommendation and greater likelihood of patient LAI acceptance. **(d)** Patient with prior history of a forced injection during a hospitalization may have more negative perceptions of LAIs and be less likely to accept LAI recommendation. **(e)** Patients who tried an LAI after a history of nonadherence may have more negative perceptions of LAIs. **(f)** Patients offered LAIs first in the outpatient setting are more likely to have more positive perceptions of LAIs. **(g)** Patients with strong support from family/caregivers or social workers/case managers may be more likely to continue treatment due to recognition of benefits. **(h)** Patients with greater trust in their outpatient HCP may have a more positive perception of LAIs.

This global, qualitative study of HCPs, patients with schizophrenia, and caregivers provides a view of treatment goals and experiences with LAIs from a variety of perspectives and sheds light upon reasons for global variability in LAI use. Strengths of the study include the multinational sampling and the use of semi-structured qualitative interview guides that were created on the basis of published evidence regarding barriers and facilitators to LAI use. Another strength of the study is the input gained from the 3 most relevant stakeholders in the schizophrenia recovery pathway: patients, caregivers, and clinicians. Findings from this work can be applied to schizophrenia recovery in global settings and have also informed a conceptual depiction of the patient journey from diagnosis through treatment selection and monitoring (**Figure 6**). Additionally, these findings have important implications for health policy.

### 4.1 HCP participants

HCPs described several factors that influence their perceptions of LAIs including availability, cost, side effect profile, patient preference, and patient care setting. Particularly in inpatient settings, psychiatrists were generally more focused on managing positive symptoms as their top treatment goal, followed by improving patient QoL. In contrast, psychiatric nurses in our sample highlighted improving patient QoL as their top treatment goal. This may relate to the type of relationship between the HCP and patient. For example, psychiatric nurses may spend more time with patients and learn more about their daily lives, resulting in this shift in focus. Overall, the HCPs in this study expressed positive perspectives on LAIs and described potential benefits of LAI use for both patients and clinicians but often indicated that they reserved LAIs for patients who were nonadherent to oral medication. The most frequently reported barriers to LAI use by HCPs in this study were patient aversion to injections, logistical challenges (e.g., lack of staff and training), and product availability and cost.

The results of the study presented here have some overlap with those in previous reports. In keeping with the observed HCP preference for reserving LAIs for patients who were nonadherent to oral medications, a survey of HCPs (*N* = 379) revealed that they may be hesitant to discuss LAI therapy with patients who are adherent to their OAs (24). In the Prevention of Relapse in Schizophrenia (PRELAPSE) study, despite positive opinions of LAIs, HCPs generally focused on the benefits of LAIs for nonadherent patients and were hesitant to recommend LAIs to younger patients (14). This hesitation occurs despite studies that have demonstrated benefits of LAIs when used early in the disease course, including reduced relapse risk (25), improved symptom control (26), and management of treatment costs (e.g., hospitalization, outpatient visits) (27). Overall, although some HCPs use LAIs early in disease treatment, LAIs continue to be underused (13).

In the clinical setting, delays in initiating LAIs despite evidence for their benefits early in the disease indicate a knowledge gap that could be mitigated through education on the potential benefits of earlier LAI treatment. Systemic barriers identified in this study such as product availability and cost are challenging for HCPs to overcome, yet barriers related to perceptions, such as beliefs that patients will reject LAIs because of fear of injections, may be addressed by educating HCPs on patient preferences related to LAIs and by encouraging discussions of patient preferences for treatment early in the disease course. Furthermore, psychiatric nurses generally reported limited awareness of specific LAI molecules, suggesting a need for additional focused education.

### 4.2 Patient and caregiver participants

The experiences and emotions of patients across their respective journeys revealed key elements that affect patient perspectives on LAIs (**Figure 6**). Interactions with HCPs, levels of family support, past hospitalizations, and logistical challenges (e.g., distance to injection clinic) all affect patient perceptions of LAIs throughout their journey. In particular, this study identified the importance of treatment setting on patients’ perspectives on LAIs. In Australia, Canada, Germany, and Israel, patients reported that the inpatient treatment discussion experience was completely driven by psychiatrists, with patients feeling like they had very little autonomy. If these patients received an LAI as part of more severe acute episodes that required inpatient care, particularly if it was received as a forced or court-ordered injection, they may associate LAIs with loss of autonomy and pain, potentially creating a barrier to future acceptance of an LAI and preference for OAs. Other possibilities include active paranoid symptoms influencing acceptance of injected medication or experience of benefit with clozapine (only available orally) for treatment-resistant schizophrenia. Conversely, patients who present in an outpatient setting early in their illness course may be more likely to be involved in their treatment decision, have greater trust in their HCPs, and engage in more discussion surrounding their QoL. Overall, patients who were offered LAIs initially in an outpatient setting are likely to have more positive perceptions of LAIs than those in inpatient settings.

During the monitoring and maintenance phase of the journey, relationships and support for the patient can affect their openness to treatment recommendations and maintenance. Patients with strong support from family, caregivers, social workers, and/or case managers may be more likely to continue treatment because of the improvement of certain patient outcomes, such as psychotic symptoms. Caregivers discussed the importance of shifting focus throughout treatment from symptom management to recovery and QoL to help patients get back to the things that they enjoy. Importantly, this study highlights that caregivers can make important contributions to the treatment decision-making process, particularly in outpatient settings and after hospital discharge, by encouraging communication. This is especially important for persons who have limited insight, a lack of motivation, and/or a lack of trust in clinicians.

Patients in this study reported convenience and fewer side effects as top advantages of LAIs in comparison with OAs. However, patients were still concerned about the potential for side effects. Caregivers acknowledged the benefits of LAIs for themselves and the person they cared for, including convenience, adherence, quicker efficacy, fewer side effects, fewer subjective reminders of disease, and reducing the number of discussions about treatment. The presence of a caregiver, a social support system, or both may even be associated with a greater likelihood of LAI recommendation by an HCP and LAI acceptance. However, caregivers also reported the need for more education and discussion about available treatments, including recognizing and reporting the convenience of LAIs to the people in their care.

The complex interplay between a patient’s treatment journey and their perception of treatments such as LAIs observed in this study is further supported by findings in the literature. Patients with schizophrenia often follow a recurring pattern of maintenance and relapse that may result in encounters with inpatient and outpatient systems and HCPs (28). Symptom presentation and the context of initial diagnosis and treatment vary and are often influenced by demographics, systemic factors, and disease severity. Systemic factors, such as availability of psychiatrists and cost of care, can lead to patients receiving care at inpatient facilities (10). With the limitations on available outpatient psychiatric care in some countries (Australia, Canada, Germany, and Israel in this study), people living with schizophrenia in these countries may see general practitioners less optimally equipped to provide schizophrenia management and treatment.

As observed in this study, it has been shown that a strong support system, combined with behavioral interventions, can help improve medication adherence (29). Our findings are also consistent with a previous qualitative study (*N* = 16) using semi-structured interviews of HCPs, patients, and caregivers in the US that identified the presence of a collaborative relationship and treatment culture between HCPs and patients/caregivers that emphasized common goals and provided respect for autonomy as an important driver of LAI acceptance (30). In addition, the STAR NETWORK Depot Study (*N* = 451), a multicenter, observational, longitudinal study in Italy, reported that improvements in patient engagement with the outpatient psychiatric service was the most common reason for prescribing LAIs and treatment adherence (31). In a non-random study of 60 HCPs using recorded HCP-patient conversations in the US, patients were more likely to accept LAIs if the HCP discussed the benefits of newer LAIs or better therapeutic effects with LAIs compared with OAs (32). Finally, as caregivers in the present study emphasized the importance of focusing treatment goals on recovery and QoL in addition to symptom reduction, a positive relationship between a patient’s QoL and severity of psychotic symptoms and the caregiver’s QoL was uncovered in an outpatient study of patients with schizophrenia in Bolivia, Chile, and Peru (*N* = 253) and their caregivers (33).

Patient education on the safety profile of LAIs and setting expectations around the potential for injection site reactions may improve patients’ acceptance of LAI recommendations. Our findings reinforce the importance of making patient and family education on drug treatments (including on LAIs) practical, readily available, and accessible in both inpatient and outpatient care settings. Potentially effective models to provide this type of education and support include the use of peer educators or peer specialists who have “been there” to help promote informed decision-making, or family-based psychotherapeutic and care coordination modalities (34, 35). With respect to health policy implications, making specialty psychiatric care and support available to individuals and families as early as possible in the schizophrenia recovery journey may help reduce some of the negative downstream complications that a substantial proportion of participants experience.

### 4.3 Limitations

The qualitative interview portion of the ADVANCE study focused on gaining an in-depth understanding of the perceptions of those involved in the care of people living with schizophrenia. However, qualitative methods have some inherent limitations. Because of the detailed and time-intensive nature of the interviews, the number of participants in each group is limited and each country is represented by a few HCPs, limiting the representativeness of country-level results. In addition, including participants from various countries, each with their unique guidelines and treatment protocols, is useful in understanding experiences across regions, but differences in practice patterns and healthcare systems introduce heterogeneity. In combination with the small sample size and potential selection bias, this may limit the generalizability of some findings. However, the variety of the vendor’s social/digital and professional sourcing channels and alignment of the participants’ background with the research objectives provides reasonable confidence that the findings from these participants are relevant to the broader populations of HCPs, patients with schizophrenia, and caregivers who provide care for individuals living with schizophrenia. This study did not include psychologists or psychotherapists, but because of the close therapeutic relationship these HCPs have with patients, their inclusion in future studies may provide additional perspectives that influence utilization of LAIs. Because participants in the study had to have experience with LAIs, these results may not be representative of individuals involved in the care of people with schizophrenia who have not used or been recommended LAIs. The type of current or past LAI treatment was not available for every patient, and the dosages of current or past LAI treatment were not collected as part of this study. More detailed information on drug type and dosing regimen was collected as part of the quantitative component of the ADVANCE study, which will be reported separately. Triads of a patient with their caregiver and their HCP were not included in the current study owing to the difficulty in identifying triads. Finally, as a survey study, results are subject to recall bias and the possibility that respondents misinterpreted the interview question.

## 5 Conclusions

Interactions between patients and psychiatrists or psychiatric nurses vary depending on the care setting, which can influence the acceptance of LAIs. The top HCP-reported treatment objectives varied by setting of care, with a primary objective of stabilizing/managing positive symptoms for inpatient care and improving patient QoL for outpatient care. HCP attitude toward LAIs is influenced by LAI availability, side effect profile, and both patient preference and HCPs’ perceptions of what patients will prefer. There is a lack of consensus about the ideal patient profile for LAIs, although most interviewed HCPs agreed that use in younger patients or patients with less severe symptoms should be limited. However, this contrasts with evidence that the use of LAIs earlier in the disease process and in younger patients may be beneficial.

Key variables during the patient journey from symptom onset to diagnosis and treatment initiation may affect the likelihood of a patient being recommended or accepting an LAI or both. The setting of initial schizophrenia presentation, family support, hospitalizations, trust in their HCP, and logistical challenges may all play a role in patient outcomes and perceptions of LAIs. Patients and caregivers acknowledge the benefits of LAIs, but they have concerns about side effects. Families and caregivers can play an important role, especially in the outpatient setting, to bridge the gap between a patient with low insight, motivation, and/or trust in their HCPs and treatment options, including LAIs. The majority of caregivers in this study were unaware of LAIs, suggesting that caregivers would benefit from educational materials to encourage conversations between HCPs, patients, and caregivers. Education on LAIs directed to the specific concerns of HCPs, patients, and caregivers may reduce barriers to LAI use.

## Data Availability

The original contributions presented in the study are included in the article/**Supplementary Material**. Further inquiries can be directed to the corresponding author.
