# Supplementary material for: Experiences and perceptions of patients, caregivers, and healthcare professionals with long-acting injectable antipsychotics for the treatment of schizophrenia: qualitative results from the multinational ADVANCE study
Source: Front Psychiatry. 2025 Dec 9;16:1645328. doi: 10.3389/fpsyt.2025.1645328 (PMC12722928; doi:10.3389/fpsyt.2025.1645328)
Supplement: Supplementary file 1 [file Supplementaryfile1.docx]

**Supplementary Materials**

**HCP Interview Questions Used in the ADVANCE Study**

**Section 1: HCP Background and Patient Population**

1. Could you please provide a brief description of your professional background and primary clinical

practice?

2. Can you provide a description of your patient population?

3. Can you talk about your professional experience as it relates to the diagnosis and treatment of

patients with schizophrenia?

4. Compared with other conditions you treat, would you say that treating patients with schizophrenia

is more or less challenging? Why?

a. Is treating schizophrenia more or less rewarding? Why?

5. In a typical month, how many patients with schizophrenia do you personally manage?

6. How do patients with schizophrenia arrive at your site of care?

a. Who are the referring clinicians?

b. Why are patients referred to you?

c. Do referred patients typically arrive with a schizophrenia diagnosis already or do you provide the

initial diagnosis in most cases?

7. Are you typically responsible for the long-term management of your patients with schizophrenia? If not, who is responsible?

a. [If HCP works in an outpatient setting] Do you have dedicated days for medication follow-up, or do

you only have days where injections are administered?

i. If you do not see patients for follow-up, who does?

b. [If HCP works in inpatient and outpatient settings] How many inpatients with schizophrenia

that you treat also come to your office for outpatient care?

8. Are there nonprescribing HCPs (e.g., social workers, NPs, RNs, PAs, pharmacists,

psychologists, clinic managers) who support your management of patients with schizophrenia? Could you describe the support network at your practice for patients with schizophrenia?

a. Can you describe their primary roles and responsibilities?

b. Are there other HCPs in your office/practice that you collaborate with for nonmedical treatment,

like psychotherapy? Do they play any role in recommending treatment?

9. What is your level of involvement, if any, in clinical studies for schizophrenia?

a. How, if at all, has this impacted your approach to managing patients with schizophrenia?

**Section 2: Treatment Approach for Schizophrenia**

10. What are the primary treatment objectives that you have for a patient with schizophrenia?

a. Does this differ by patient profile?

b. Do your treatment objectives differ depending on the type of antipsychotic you are prescribing?

c. Do you ever first use first-generation antipsychotics (FGAs)? Why? How often do use them?

11. Describe your evaluation and treatment approach for your patients with schizophrenia.

a. What has influenced your treatment approach?

i. Do you follow any practice guidelines? If so, which organizations?

b. Do you have the opportunity to discuss schizophrenia cases with your peers or other members of

your treatment team? If yes, how helpful are those discussions and why? If not, what is the barrier?

c. If you had a particularly difficult case or were weighing the pros and cons between treatment

options, would you seek guidance? If so, what resource would you use for guidance?

d. Do you attend continuing medical education (CME) or other peer-to-peer events? If yes, how do

these inform your treatment approach? If no, why not?

e. Has your treatment approach changed over time? If so, why?

f. Have you ever used clozapine as a treatment option in your practice?

12. What proportion of your patients are nonadherent to antipsychotic medication? To clinic visits?

a. Could you describe typical patient characteristics that you would associate with nonadherence (or

the risk of nonadherence)?

b. Does your approach change for nonadherent patients? How so?

i. Do you ever voluntarily or involuntarily consider going to the court or suggest guardianship to

the family?

1. [If yes to going to court] Do you seek involuntary treatment and if so, does that occur in an outpatient or inpatient setting? Does patient adherence improve after involuntary treatment?

13. Can you describe how you typically discuss treatment options with your patients with schizophrenia?

a. What types of strategies are used to discuss and present treatment options?

i. Does this differ by patient?

b. Who else is involved in these discussions?

i. What level of influence do these stakeholders have on your recommendation when it comes to

treatment?

14. Would you say their perspectives on treatment are similar to those of the patient? Why or why not?

a. What happens in cases where there is not alignment between the patient and other

stakeholders?

ii. What level of influence do they have on the patient’s decision?

**Section 3: Experience/Perceptions of LAIs**

14. What are your perspectives on LAI antipsychotics for schizophrenia? Do you have a generally positive

or negative opinion on LAIs for patients with schizophrenia?

a. Can you describe a positive experience you had with LAIs? What was the outcome?

b. Can you describe a negative experience you had with LAIs? What was the outcome?

c. What product attributes are most important to you when considering a treatment option for

schizophrenia?

i. How do you think LAI product attributes affect your conversation with patients?

ii. Which attributes make you more likely to offer an LAI? Less likely?

iii. Which attributes makes the conversation easier, and which do you think help patients accept LAIs?

d. What would improve your perception of LAIs? What types of information might lead you to think

more positively about LAIs?

e. How often do you mention/introduce/discuss LAIs with your patients with schizophrenia? How often do you recommend an LAI for your patients with schizophrenia?

i. Do you ever have patients (or family members) that ask to be put on an LAI?

f. What proportion of your patients started on an LAI as an inpatient vs outpatient?

g. What are your perspectives on using LAIs for outpatients vs inpatients?

h. Does the availability of an analogous LAI formulation factor into your decision of what oral antipsychotic to prescribe a patient?

i. In cases where a patient is prescribed an oral antipsychotic for which there is not an LAI formulation, how often does the patient eventually try an LAI? (Or does this remain a barrier to transitioning to an LAI)?

i. Has your perspective and/or use of LAIs changed over time? Why or why not?

i. What contributed to your change of perspective or lack thereof?

ii. What were you taught regarding LAIs for schizophrenia during your training? How much has your training informed your perspective and strategy of using LAIs for schizophrenia?

iii. How often do you discuss LAIs with peers? Have these conversations changed your perspective/use of LAIs? Why?

15. When you prescribe an LAI for schizophrenia treatment, how confident are you that you will get the

results you hope for?

a. In what cases are you more confident? Why?

b. In what cases are you less confident? Why?

16. At what stages in the schizophrenia disease course would you prescribe an LAI (e.g., first-episode

psychosis, maintenance, relapse, nonadherence, etc.)? Why?

a. What does successful treatment look like for patients at each stage of their illness?

i. Are your treatment goals aligned with those of the patient?

b. Could you give me an example of a patient that you would prescribe an LAI to at each stage in the

schizophrenia disease course?

17. What are the typical clinical characteristics in a patient with schizophrenia that would compel you to

recommend an LAI?

a. Do patient demographics differ between those typically on LAIs and those typically on oral antipsychotics? Why?

b. Is there general alignment between the patients you feel would most benefit from an LAI and

those patients who are most likely to accept them? Why or why not?

18. How do you approach treatment discussions when an LAI is involved? Could you walk me through

how a typical discussion about LAIs goes with your patients with schizophrenia?

a. What information do you share with the patients?

i. Do you ever show posters or manuscripts showing scientific data on advantages of LAIs

compared with oral antipsychotics? Why or why not? If so, how do you present it?

ii. Do other members of your practice share information with the patient?

b. Are there some patients where education on LAIs takes longer than others? For which patients is

that a challenge?

c. What questions do patients ask? How well informed are they?

d. What questions do caregivers/family ask? How well informed are they?

e. How do you choose which LAI to recommend?

19. Tell me about a time that you strongly recommended an LAI to a patient with schizophrenia.

a. How typical is it for you to strongly recommend an LAI to a patient with schizophrenia? Why?

b. What patient characteristics compelled you to strongly recommend an LAI?

i. What would make you hesitant to recommend an LAI to a patient?

c. Which LAI did you recommend and why?

d. What did you tell the patient during the discussion?

e. How did the patient respond? Did they accept your recommendation?

i. What questions did they ask?

f. Were any other people involved in the discussion (nurses, social worker, caregiver, family, other

HCPs)? What impact did they have on you or your patient?

i. How does your relationship with a patient impact your recommendation and strength of

recommendation? What is the role of trust in your decision to recommend an LAI?

20. Describe a conversation about LAIs that went really well. Why did it go well?

a. How often do your conversations about LAIs go well like the example you provided?

21. Describe a typical conversation about LAIs that didn’t go well. Why did it not go well?

a. When faced with a hesitant patient, how do you give them confidence in an LAI?

b. Do you ever continue to recommend an LAI even after a patient has rejected your recommendation? Why or why not?

i. Can you briefly give an example of a time where you left the decision up to the patient?

ii. Have you ever been forced to give an LAI to patient due to court order or as part of involuntary

treatment? What was that experience like?

22. How do you feel when you switch a patient onto LAIs? Positive, negative, or neutral? Why?

a. Do you have any hesitancies or concerns when deciding to put a patient on an LAI?

23. What do you think patients like most about LAIs? What do you think they dislike the most?

a. What do you believe are the key reasons a patient would ***choose*** to try an LAI?

b. What do you believe are the key reasons a patient would ***refuse*** to try an LAI?

24. Are there nonclinical patient characteristics that influence your decision to recommend/prescribe

an LAI?

a. Are there LAIs that you do not mention/recommend due to previous issues with cost and market

access?

25. Are there logistical considerations at your practice that impact your decision to

recommend/prescribe an LAI?

a. How does the availability of injection nurses factor into your decision to prescribe LAIs?

b. Are there some patients where education on LAIs takes longer than others? For which patients is

that a challenge?

26. Are there specific LAI product characteristics that would increase your likelihood to recommend LAIs

to your patients with schizophrenia? That would decrease your likelihood to recommend LAIs?

a. If you could describe your “ideal” LAI, what would it be?

i. Would you be more or less likely to offer this “ideal LAI”? Why?

ii. Would the conversation about this "ideal LAI” be easier/less stressful? Why?

iii. Do you think patients would be more willing to accept/try this “ideal LAI”?

27. How important are drug manufacturer–provided patient support programs? How do they influence

your decision to prescribe LAIs?

a. Which specific programs have your patients had the best experience with/support from?

**Patient Interview Questions Used in the ADVANCE Study**

**Section 1: Introduction and History With Schizophrenia**

1. Can you tell me a little about yourself?
   1. How old are you?
   2. Do you live in an urban, suburban, or rural location?
   3. What is your current housing situation?
   4. What is your current employment status?
   5. Do you have health insurance? If so, what kind?
   6. How do you spend your time?
      1. What hobbies do you partake in?
      2. Who do you spend your time with?
   7. Do you have any other diagnosed mental health conditions such as depression, anxiety, bipolar disorder, OCD?
2. Now, I’d like to spend a few minutes hearing about when you were first diagnosed with schizophrenia. How long ago were you diagnosed/what age were you when you were diagnosed?
   1. Did you experience symptoms or realize something was different and seek help/treatment on your own?
      1. If not, did a family member or caregiver notice symptoms and recommend you seek treatment?
   2. What symptoms were you experiencing when you were diagnosed?
   3. How long had you been experiencing symptoms before you were diagnosed?
   4. What type of doctor/clinician made the diagnosis?
   5. Was the diagnosis made in the hospital or outpatient setting?
   6. Thinking back to that time, what were you told about the condition?
      1. What was your reaction to having this diagnosis?
      2. Did you know anyone who had this condition before and/or if did you know anyone who has received treatment for this condition?
      3. Did you perceive any stigma associated with the diagnosis and treatment of schizophrenia?
   7. Did you interact with anyone else from the hospital team during this time?
3. How long after your diagnosis of schizophrenia did you receive medication?
   1. What medication were you first recommended?
      1. Is that the medication you eventually received? Why or why not?
   2. What were you told about the treatment options? How was the decision made to choose that medication option?
      1. Did your doctor recommend a specific medication or were a range of options presented to you to choose from?
      2. Did anyone else discuss medication options with you?
      3. Did you accept the first medication option that was recommended to you? Why or why not?
   3. How familiar were you with the medication before you began taking it?
      1. How much did you research the medication? What were the sources of information you were looking at?
      2. What did you learn about the medication and what were your initial perceptions of it?
   4. What were you hoping to gain from taking the medication? Did you have any initial concerns about the medication?
      1. For how long were you on that medication?
      2. Did you use the medication exactly as prescribed?
      3. How satisfied were you with the medication?
      4. Did your experience match your initial perceptions of the medication?
4. What other medications have you taken since then to treat your schizophrenia?
   1. Have your expectations towards these medications changed over time? Why?
5. Have you ever gone to the hospital due to your schizophrenia? Could you describe what that experience was like?
   1. What was your experience when you were discharged? Did your perspectives towards your condition or medication change as a result?
   2. Were you ever re-hospitalized? What do you think led you to be re-hospitalized?

**Section 2: Current Experience With Schizophrenia**

1. What are your current goals for managing your schizophrenia condition?
2. What role does your doctor play in helping you achieve your goals?
3. What role does medication play in helping you achieve your goals?
4. How well do you feel your schizophrenia is currently managed?
   1. What are your biggest challenges?
   2. What do you think would improve your ability to manage your schizophrenia?
   3. How do you feel about taking medications for schizophrenia? Please consider moments when you feel comfortable and positive about taking medications, as well as times when you may lack motivation or feel that the burden outweighs the benefit.
5. Describe your relationship with the doctor who treats your schizophrenia.
   1. Do you trust your doctor or prescribing clinician? Why or why not?
   2. Do you feel that your doctor listens to you? Why or why not?
   3. Are you being treated by the same doctor who diagnosed you? If not, what led you to be treated by a different doctor?
6. Are there other healthcare professionals (e.g., nurses, social workers, etc.) who have been involved in discussions about medication options?
   1. Can you describe these individuals and the nature of your relationships with them?
   2. What role have they played in discussions around treatment options?
7. Do you have a caregiver/family member or friend who participates in the discussions or whom you talk to about what the doctor says?
   1. Are there any other channels of information and support available for you (e.g., patient organizations, clinic meetings, online sites/forums)?
   2. Do you have anyone in your family or social network who advises you not to take medications?
8. Think about the last time you talked to your doctor about new medications or treatment options for your schizophrenia. Describe how that conversation went.
   1. What was the most important thing you considered?
   2. Were there considerations that led you to reject certain medications as an option for you?
   3. Did you end up making any compromises?
9. Can you give me an example of a time where somebody had a significant influence over your treatment decision?
10. Has your doctor ever recommended a medication for your schizophrenia you weren’t comfortable with (at least at first)?
    1. What medication was it?
    2. Why were you uncomfortable with it?
    3. What did the doctor do when you expressed your discomfort?
    4. Was your caregiver or other healthcare professionals (nurse/support staff) involved in the conversation?

**Section 3: Experience/Perceptions of LAIs**

1. What word or phrase first comes to mind when you think of LAIs? Explain.
2. When did you first hear about LAIs? What were your initial perceptions of them?
3. When were you first recommended an LAI? What were you told about them?
   1. Who first recommended an LAI to you?
   2. Why did they recommend it to you?
      1. How strong was their recommendation?
   3. What questions did you have?
   4. Where did you go to get them answered?
   5. What information did you find most useful?
4. When LAIs were first recommended to you for your schizophrenia, what advantages did you think they may offer you compared with an oral medication?
   1. Which advantages were most important to you?
   2. How did/do you think they might make your life better?
5. What concerns did you have? Which concerns were most important to you?
   1. Did you express these concerns to your doctor? Were they addressed by the doctor? How were they addressed?
6. Did you talk to other people about LAIs?
   1. What did they think would be advantages/disadvantages of taking LAIs?
   2. How much influence did they have on your decision?
   3. Have you ever spoke to peers who have also tried LAIs?
      1. If so, how did those conversations impact you and your perspective on LAIs?
      2. If not, would you like to speak with someone who has also tried an LAI? Why or why not?
7. Did you look for information online?
   1. Where did you look?
   2. What information did you find?
8. When you were first recommended an LAI, did you agree to try it? Why or why not?
   1. *[If no]* Have you ever tried an LAI since then? *[If no to 23a]* Would you ever consider trying an LAI in the future? Why or why not?
   2. Do you know anyone else who takes an LAI for treatment of their schizophrenia?
9. *[If they tried an LAI]* How long did it take from when the doctor recommended LAIs to you agreeing to try them?
   1. Why did it take a short/long time?
   2. What did you do during that time? Why did you ultimately agree to try it?
10. When you agreed to try an LAI, what did you hope it would do for you?
11. Can you describe your experience with LAIs?
    1. What benefits did you observe?
    2. What challenges did you face?
    3. How satisfied were you with the medication? What would have improved your experience?
       1. Do you have any regret deciding to try an LAI?
       2. Do you have any regrets deciding not to try an LAI sooner?
    4. Have you tried more than one type of LAI?
12. Are you currently on an LAI?
    1. Why did you decide to stop? Was it at your doctor’s recommendation or your decision or both?
    2. What, if anything, would lead you to reconsider an LAI?
13. How did your experience taking an LAI impact your relationship with your doctor?
    1. Did it impact the level of trust you placed in your doctor?
    2. How did this change your level of involvement in future choices of medication?
14. How did your experience taking an LAI influence your perspective on the healthcare system, in general?
    1. What were the main challenges you faced obtaining LAIs?
    2. Can you give me an example of something that worked very well? How did that benefit you?
    3. Can you give me an example of something that needs to be improved? How do you think it could be improved?

**Section 4: Final Thoughts**

1. Imagine someone who has schizophrenia is seeking your advice. What would you tell them is the most important thing to understand about the condition? About LAIs?

**Caregiver Interview Questions Used in the ADVANCE Study**

**Section 1: Introduction and History With Schizophrenia**

**In this first section, we would like to learn about your patient and your relationship with them.**

1. Can you tell me about yourself?
2. Can you tell me a little about the person with schizophrenia whom you care for?
   1. What is your relationship to them?
      1. Do you live with them?
   2. Do they live in an urban, suburban, or rural setting?
   3. How long ago were they diagnosed with schizophrenia?
   4. How long have you been their caregiver?
   5. What symptoms have they experienced?
   6. What types of conversations do you have with them about their schizophrenia?
      1. What do you typically talk about?
      2. Who typically initiates the conversations?
      3. Are there any topics that you avoid? Why?
   7. What do you believe are their biggest needs related to their schizophrenia?
      1. Do these align with what they believe are their biggest needs?
      2. Do these align with what the doctor believes are their biggest needs?
   8. In what ways do you support them?
3. How has their experience with schizophrenia impacted you?
4. Were you present when they were first diagnosed with schizophrenia?
   1. What type of doctor diagnosed them?
      1. Were they diagnosed in a hospital or outpatient setting?
      2. [If hospital] Were they admitted voluntarily?
   2. Who else at the hospital did you or the person you care for interact with during this time?
   3. [If yes] What role did you play during initial diagnosis?
      1. What type of support did you provide for them at this time?
5. Are they still being managed by the doctor who initially diagnosed them?
   1. [If not being managed by same doctor] What led them to be seen by different doctors? Did you ever suggest they see a different doctor? Why or why not?
   2. Has their experience with schizophrenia been impacted due to being managed by different doctors? If yes, in what way?
6. What antipsychotic treatments have they taken?
   1. [If multiple] Why did they switch or discontinue medications?
   2. Which treatments have they had the most positive experience with? Most negative experience? Why?
7. Please describe your role in the treatment decision-making process for the person with schizophrenia whom you care for.
   1. Are you present for doctor appointments?
   2. Do you discuss treatment options with the doctor? With other healthcare professionals?
   3. Do you discuss treatment options with them?
      1. What are those conversations like?
      2. What happens when you both have different opinions on treatment options?
   4. How much autonomy do they desire when making treatment decisions?
      1. How much autonomy do you feel they should have? Why?
   5. Are you responsible for making sure they take their medication?
      1. Does this lead to any conflict between you and them?
   6. Do you proactively research treatment options for them?
      1. [If yes] What sources do you rely on for learn about treatment options (e.g., social media, peers, internet, drug company materials)?
8. What treatment goals do you have for the person with schizophrenia you care for?
   1. Do you feel that this aligns with their own goals?
   2. Do you feel that this aligns with the goals of their doctor?
9. How has your relationship with the person you care for changed over time?
   1. Have their needs changed? Why or why not?
   2. Has the type of support you provide for them changed? Why or why not?
      1. How common is it for them to ask for support if they need it?
      2. Over time, have you become more confident in your ability to care for them? Why or why not?
   3. Can you describe a time where you made a positive impact in their life as their caregiver? What did you do and how did it impact them?
   4. Has there ever been a time where you offered support to them, but they rejected your offer?
      1. Why do you think they responded this way? How was the conflict resolved?
   5. Do you anticipate the need for your support for them in the future? Why?
10. What kinds of support do you need as a caregiver of a person with schizophrenia? Are those support needs met and by who?

**Section 3: Experience/Perceptions of LAIs**

1. What word or phrase first comes to mind when you think of LAIs? Explain.
2. How familiar were you with LAIs before the person you care for was first recommended them?
   1. How much did you research the medication?
   2. What did you learn about the medication and what were your initial perceptions of it?
3. When was the person you care for first recommended LAIs? What was your perception of it and what were you told?
   1. What questions did you or the person you care for have? Where did you go to get them answered?
4. What do you believe are key advantages of LAI? Which of these advantages do you think is most important to the person you care for?
   1. How, if at all, do you think they have made the person you care for’s life better?
5. What concerns, if any, did you have about LAIs?
   1. Which concerns do you think are most important to the person you care for?
   2. Are your concerns generally the same as those of the person you care for? If so, did you do anything to alleviate their concerns?
   3. Were your concerns addressed and if so, how?
6. When the person you care for was first recommended an LAI, did they agree to try it? Why or why not?
   1. What was your role in the decision-making process?
   2. Did you try to convince them to try an LAI? Why or why not?
   3. What strategies, if any, did you pursue to convince them it might beneficial?
      1. What strategies would you recommend to a caregiver who thinks an LAI would be beneficial for the person they care for who has expressed hesitancy?
   4. [If they initially refused] Did they ever decide to try an LAI at a later point? If so, why did they change their mind?
   5. Do you think they ever wished they had switched to an LAI earlier?
7. Have you talked to the person you care for about LAIs?
   1. What discussions have you had? What were the key topics?
   2. What information, if any, did you share with them?
   3. How much influence did you have on their decision to try or refuse LAIs?
8. [If their patient tried an LAI] Can you describe the experience the person you care for has had with LAIs?
   1. What benefits did you observe?
   2. What challenges did they face?
   3. How satisfied were they with the medication? What do you think could have improved their experience?
   4. Have they tried more than one type of LAI?
9. What are the top things that would improve the experience for patients with schizophrenia?

**Section 4: Final Thoughts**

1. Imagine you are giving advice to someone else who is a caregiver for a person with schizophrenia. What would you tell them?
